# Supplementary material for: Nanoscale deformation mechanics reveal resilience in nacre of Pinna nobilis shell
Source: Nat Commun. 2019 Oct 23;10:4822. doi: 10.1038/s41467-019-12743-z (PMC6811596; doi:10.1038/s41467-019-12743-z)
Supplement: Supplementary file 1 — Supplementary Information [file 41467_2019_12743_MOESM1_ESM.pdf]

# Supplemental Material: Nanoscale deformation mechanics reveal resilience in nacre of *Pinna nobilis* shell

Jiseok Gim[1], Noah Schnitzer[1], Laura M. Otter[2], Yuchi Cui[1], Sébastien Motreuil[3], Frédéric Marin[3], Stephan E. Wolf[4,5], Dorrit E. Jacob[2], Amit Misra[1], Robert Hovden[1,6]*

1. Department of Materials Science & Engineering, University of Michigan, Ann Arbor, MI, USA
2. Department of Earth and Planetary Sciences, Macquarie University, Sydney, Australia
3. Laboratoire Biogéosciences, Université de Bourgogne Franche-Comté (UBFC), Dijon, France
4. Department of Materials Science & Engineering, Friedrich-Alexander-University Erlangen-Nürnberg, (FAU), Erlangen, Germany
5. Interdisciplinary Center for Functional Particle Systems (FPS), Friedrich-Alexander University Erlangen-Nürnberg (FAU), Erlangen, Germany.
6. Applied Physics Program, University of Michigan, Ann Arbor, MI, USA

*Correspondence and requests for materials should be addressed to R.H. (email: hovden@umich.edu).

## I. Crystal Structure of Inorganic Bridges

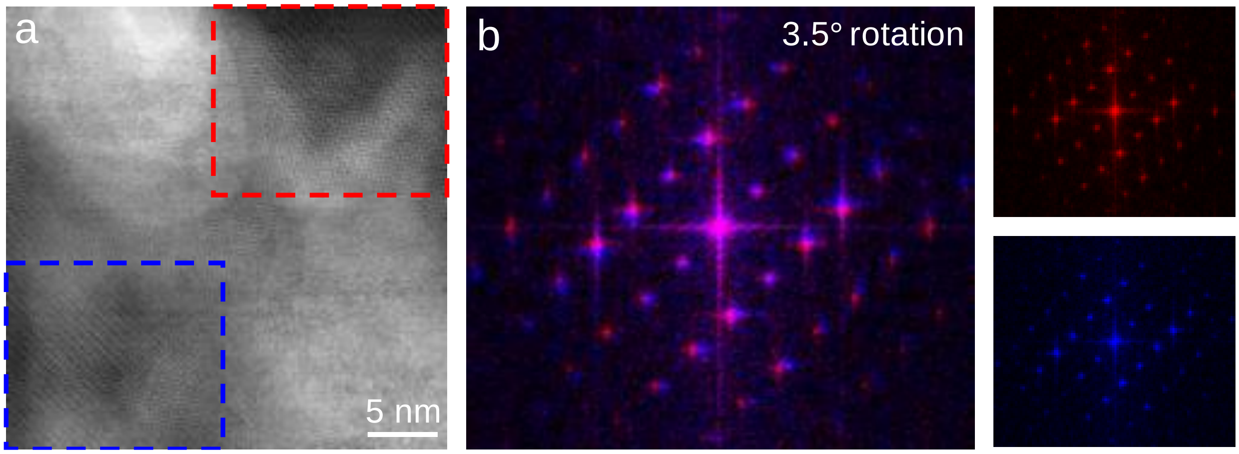


**Supplementary Figure 1.** Shared crystal orientation between two adjacent nacre tablets. a. Cross-sectional bright field STEM of a minor connection i.e. intrinsic mineral bridge structure without external stress spanning across the interlamellar membrane showing continuous crystal growth between tablets. b. Fast Fourier Transformation of (a) showing a twist angle of 3.5 degrees between the crystallographic orientations of the tablets. This could suggest continuous growth of the tablets from one layer to the next through intrinsic bridges, noted as a possible nacre growth mechanism[1,2].

## II. Quantifying Organic Concentration from HAADF Intensity

Relative organic concentration in the nacre tablets was formulated by ratio of high-angle elastic electron scattering intensity, which is defined by $I_{\mathrm{HAADF}} = t \cdot \sum Z_x^{\gamma} \cdot \rho$ where $t$ is the thickness of nacre, $\rho$ is the density of the $CaCO_3$ or an organic molecule, and $Z_x$ is the atomic number of an element in $CaCO_3$ or an organic molecule. Chitin[3,4] and lustrin[5,6] are considered as representatives of the organics in nacre because the two proteins are the main components of the matrix protein in nacre of mollusk shells, which consists of amino groups. The collected HAADF signal scales as $Z^{\gamma}$ and gamma ranges from 2 for Rutherford scattering at very large angles to 4/3 as described by Lenz-Wentzel expressions for a range of scattering angles from a screened coulomb potential. Typically, $\gamma$ is around 1.7, and this value was chosen for this calculation[7]. There is little thickness variation across the cropped 500 nm by 500 nm region of interest, equivalent to the width of the nacre tablets inspected. The intensity measured over vacuum is subtracted from the HAADF intensities and set to a positive value to avoid negative counts, then downsampled by a factor of 2 to improve signal-to-noise ratio. Total error of organic material fraction is calculated by different threshold values for HAADF intensity of 100% $CaCO_3$. The volume fraction of organics are converted into the weight fraction by multiplying the density of generic protein (1.35 $g/cm^3$)[8] and aragonite (2.95 $g/cm^3$)[9]. The values in the manuscript are averaged from two different region in nacre.

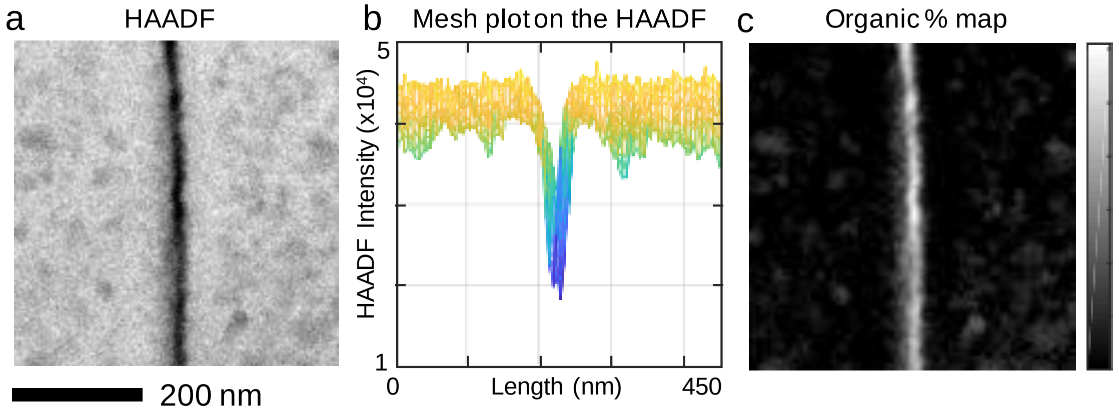


**Supplementary figure 2.** Relative organic concentration in nacre tablets using the ratio of high-angle elastic electron scattering intensity. a. HAADF-STEM showing the overview of the nacreous tablet. The region of interest is cropped to an area of 500 nm by 500 nm, which is equivalent to the width of a nacre tablet. b. Mesh plot on HAADF image in a showing the region interest has little thickness variation. c. Relative organic component concentration map on the region in a. In this example, organic materials occupy 6.9 ± 1.8 vol. % (3.3 ± 0.8 wt. %) of nacre composed of 3.0 ± 0.5 vol. % (1.4 ± 0.2 wt. %) interlamellar and 3.9 ± 1.3 vol. % (1.9 ± 0.6 wt. %) interorganic.

## III. Deformation and Recovery of Nacre Tablets

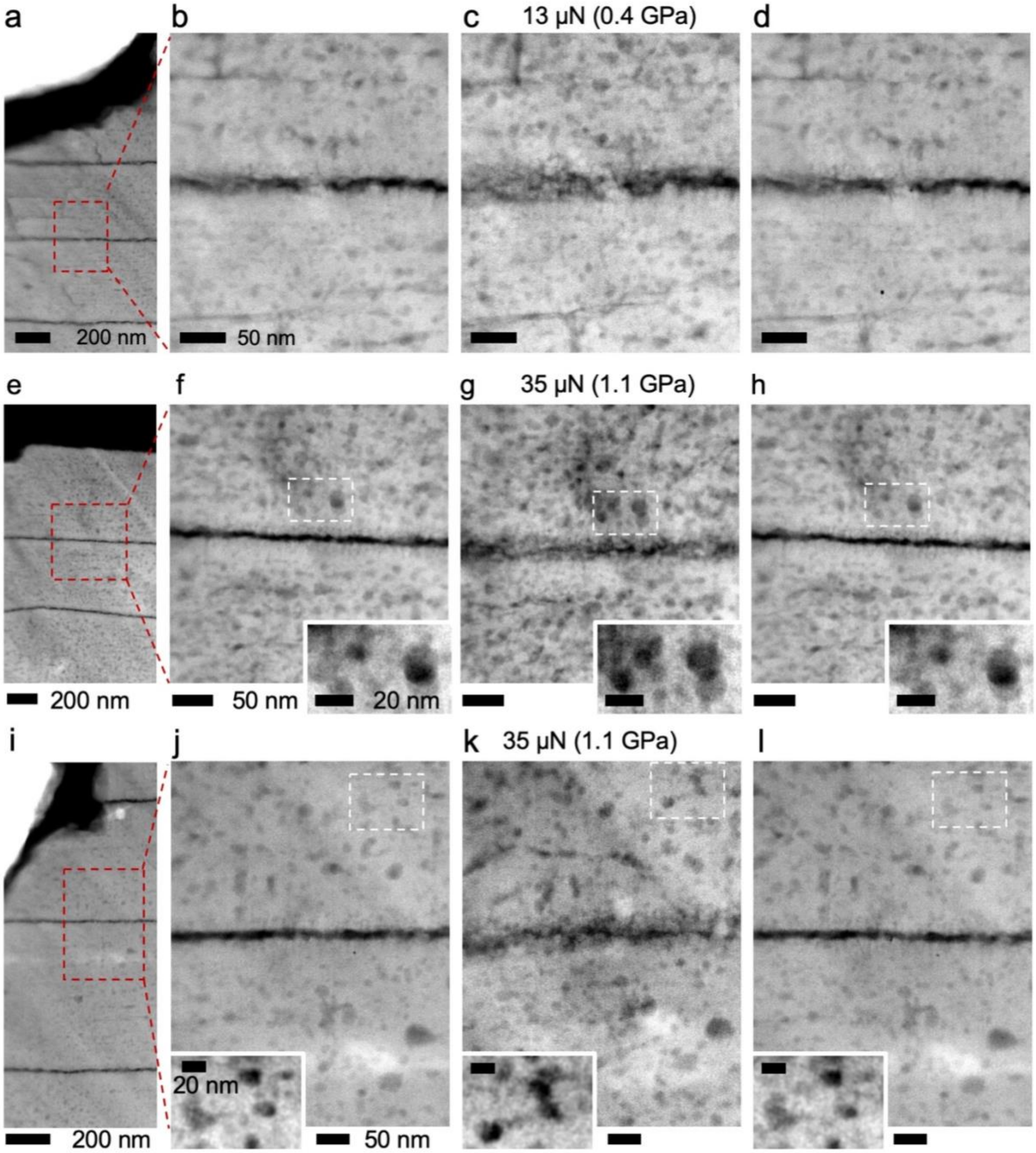


**Supplementary Figure 3.** Cross-sectional view of nacre deformation and recovery at tablet interfaces using HAADF STEM. a, e, i) Overview of three different nacre tablets. b, f, g) The interlamellar membrane between opposite tablets is well defined before compressive nanoindentation. c, g, k) Upon loading, interface interlocks and temporary inorganic connections form. d, h, l) After the load is released, the system fully recovers to its initial state. Insets highlight deformation of aragonite grains and organic inclusions as viewed in projection.

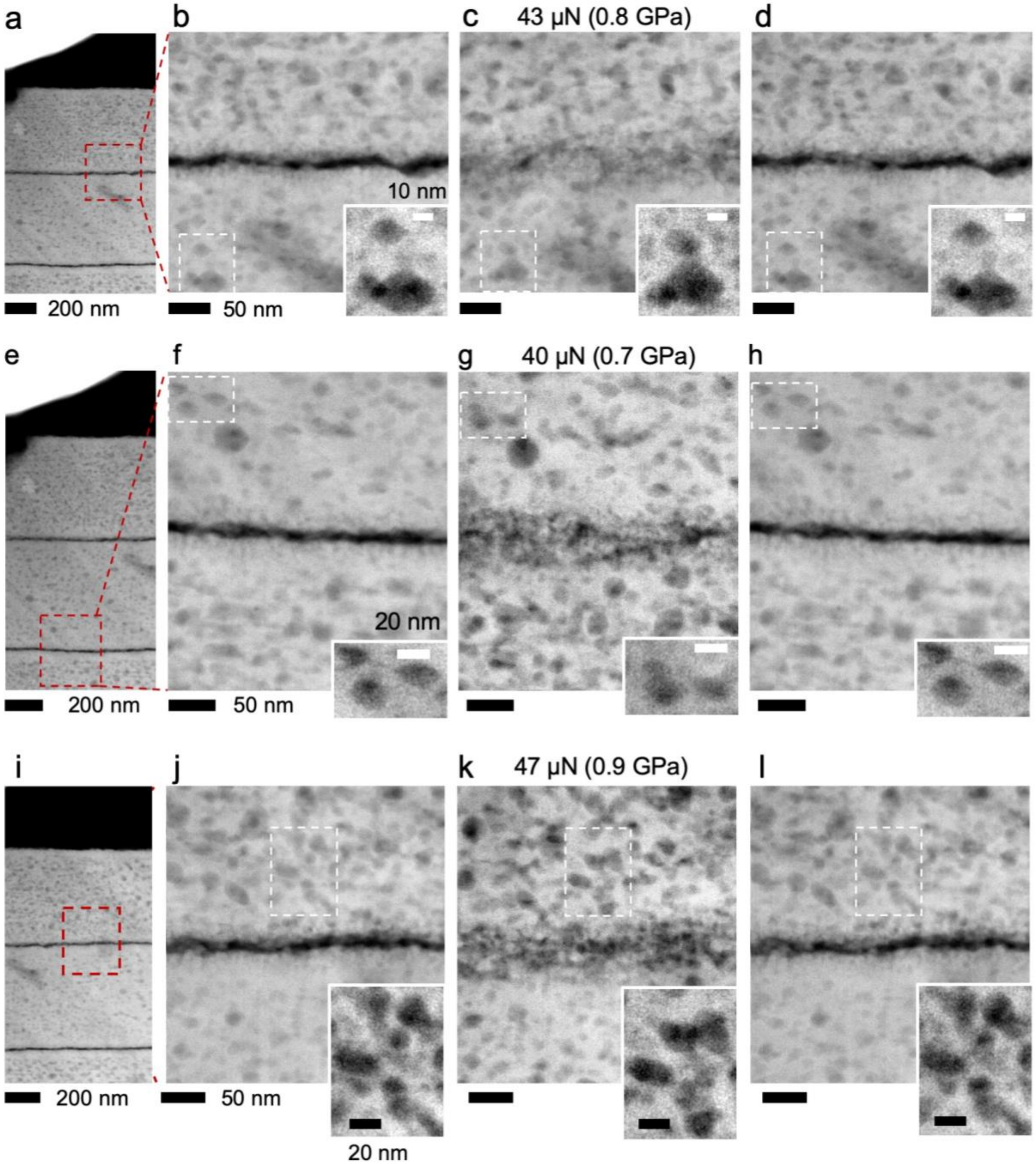


**Supplementary Figure 4.** Cross-sectional view of nacre deformation and recovery at tablet interfaces using HAADF STEM. a, e, i) Overview of nacre tablets. b, f, g) The interlamellar membrane between tablets is well defined before compressive nanoindentation. c, g, k) Upon loading, interface interlocks and temporary inorganic connections form. d, h, l) After the load is released, the system fully recovers to its initial state. Insets highlight deformation of aragonite grains and organic inclusions as viewed in projection.

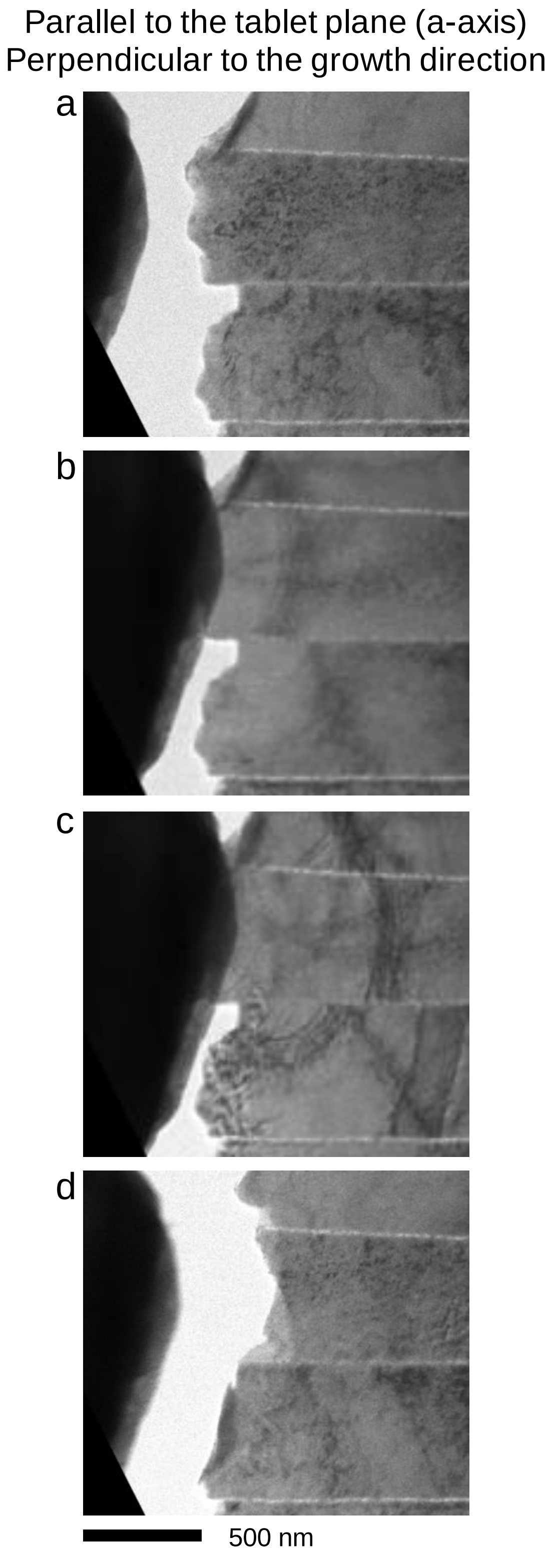


**Supplementary Figure 5.** In situ TEM nanoindentation along a-axis of nacre tablets. (a-d) Compression parallel to the tablet plane showing a limited amount of locking at interface before fracture.

## IV. Strain Attenuation in Nacre

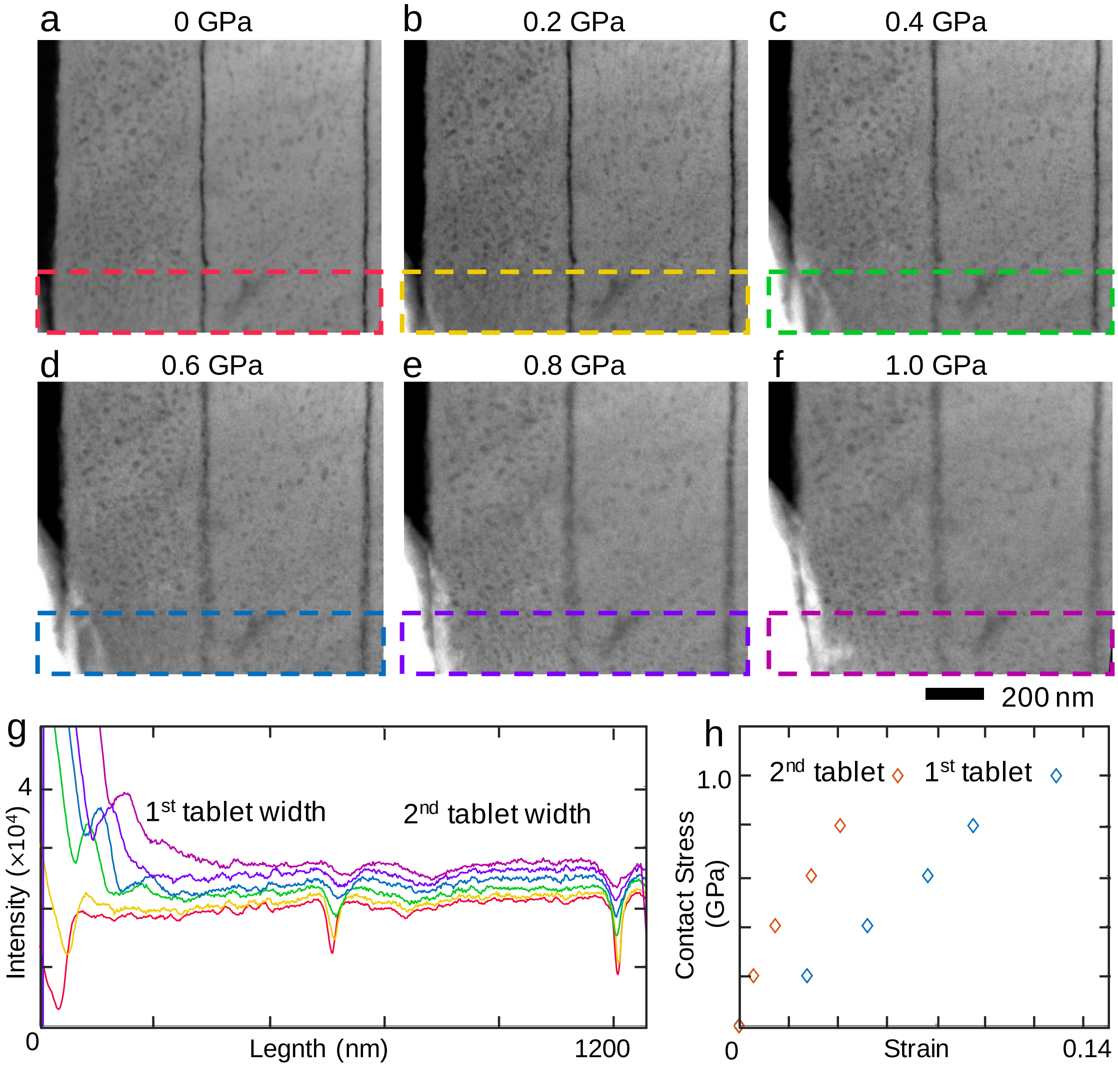


**Supplementary Figure 6.** Strain attenuation from 1st to 2nd tablet from indenter tip. a-f. HAADF-STEM images showing nacreous tablet compressed from 0 GPa to 1.0 GPa. g. HAADF intensity profiles of the cropped area in a-f showing the gradual reduction of the tablet width. h. Contact stress-tablet engineering strain curve plotted based on the ratio of 1st and 2nd tablet width measured in g.

## V. Rotation and deformation of nanograins and crack blunting

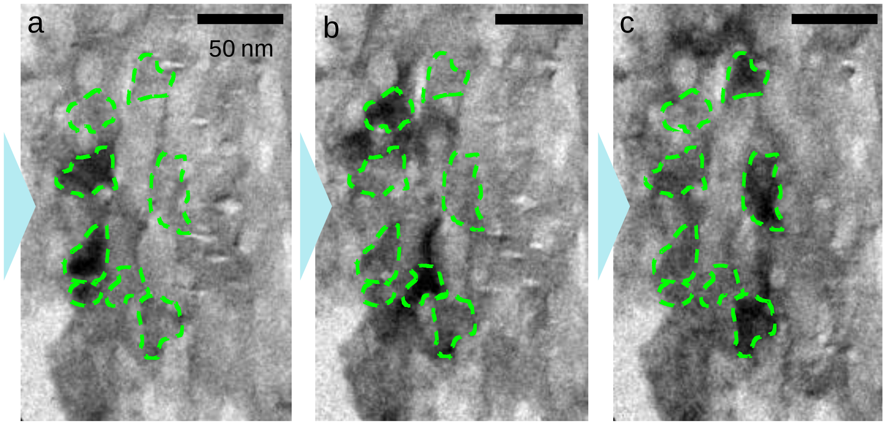


**Supplementary Figure 7.** Rotation and deformation of aragonite nanograins and organic inclusions in nacre at the nano-scale. a-c. Bright field TEM of the cross-sectional nacreous region after contact with indenter tip (noted by cyan arrow) and continuous compression of the same region. Darkening of grains corresponds to changes in crystallographic orientation. Notably, the organic inclusions of c) have become rounded in morphology compared to a, b. Here, BF-TEM contrast of the thin specimen is sensitive to strain and small changes in crystallographic orientation. The visible darkening of grains (green circles) during indentation are due to nanograin deformation or reorientation from local stresses. The nanogranular response and dynamics are most visible in the Supplemental Movie 1.

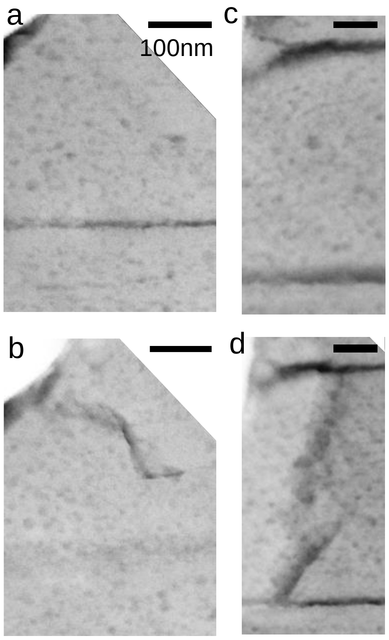


**Supplementary Figure 8.** Cracks blunted by organic boundaries and inclusions in nacre. a, c,) Nacre structure before compression. After failure cracks propagate and terminate at organic inclusions (b) or terminate at the interlamellar membrane (d).

## VI. Summary of In-Situ Movie Data

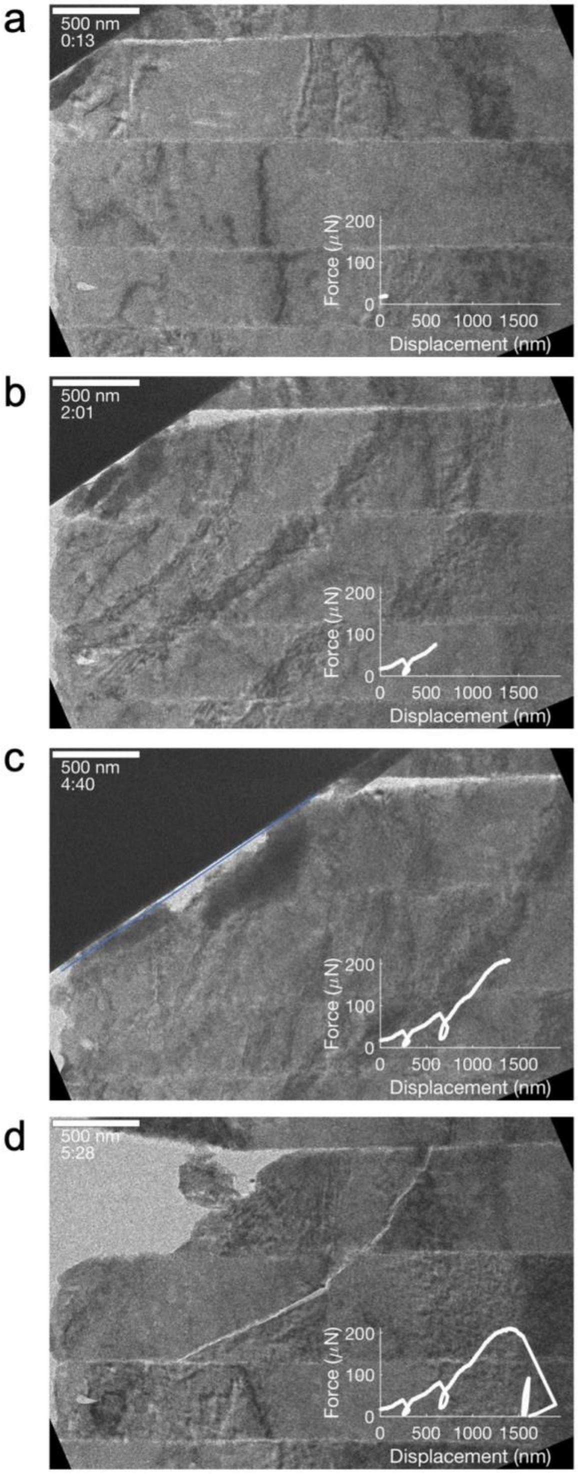


**Supplementary Figure 9.** Video stills of nacreous aragonite extracted from an *in-situ* BF-TEM nanoindentation using a cube corner diamond probe tip. Right-bottom graph in each image are the correlative load–displacement curves. Video content is included as Supplementary Movie 2.

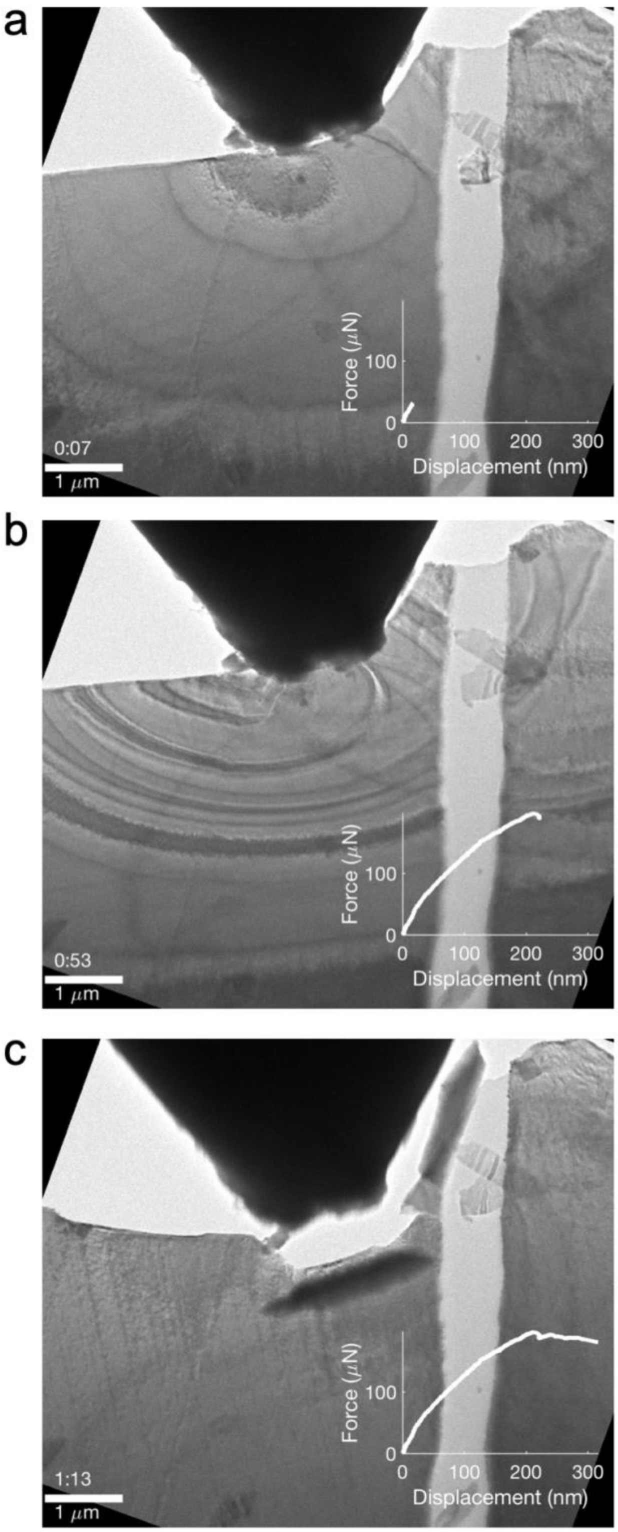


**Supplementary Figure 10.** Video stills of prismatic calcite extracted from an *in-situ* BF-TEM nanoindentation using a conospherical diamond probe tip. Right-bottom graph in each image are the correlative load–displacement curves. Video content is included as Supplementary Movie 3.

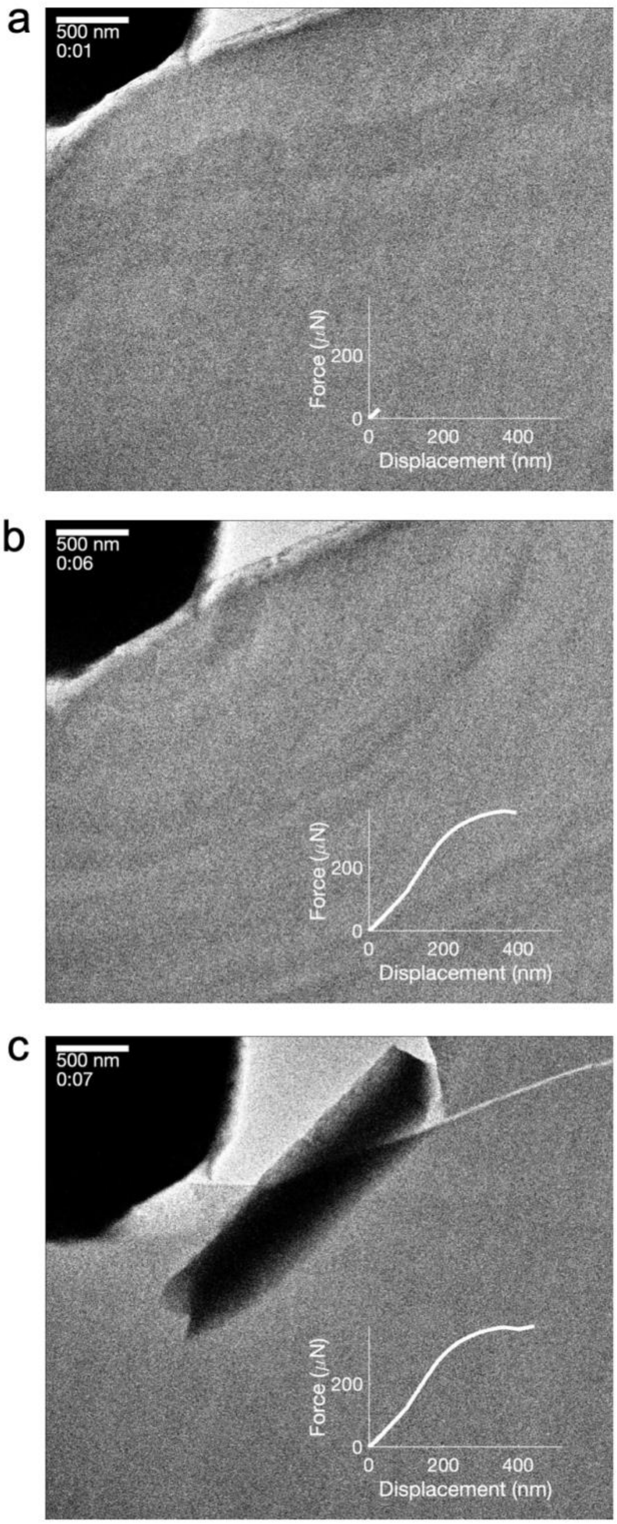


**Supplementary Figure 11.** Video stills of geological monolithic aragonite extracted from an *in-situ* BF-TEM nanoindentation using a conospherical diamond probe tip. Right-bottom graphs in each image are the correlative load–displacement curves. Video content is included as Supplementary Movie 4.

## VII. Comparison of mechanical properties on the cross-sectional and bulk specimen of nacre, prismatic calcite, and geological monolithic aragonite

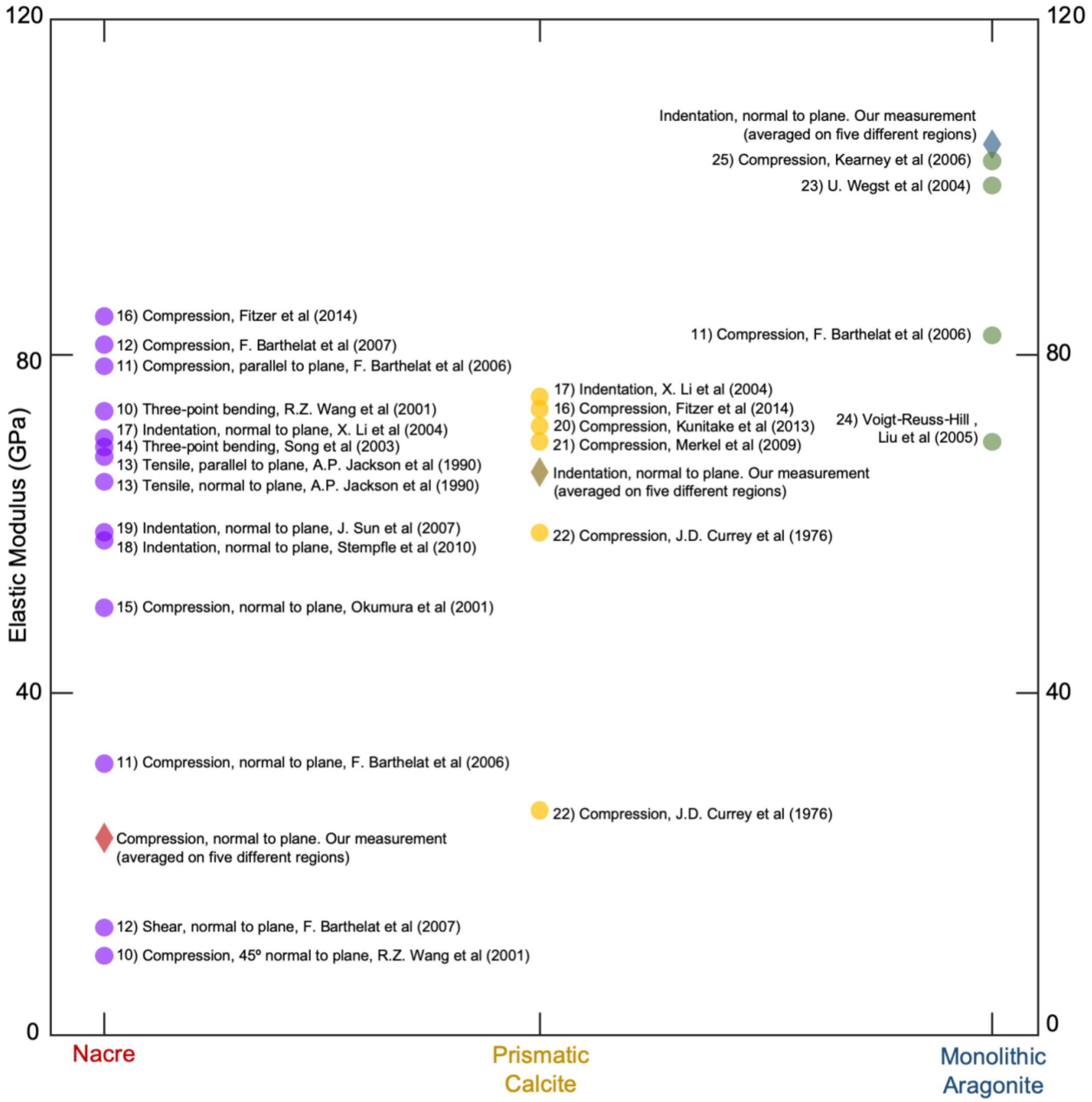


**Supplementary Figure 12.** Literature survey of elastic modulus of nacre, prismatic calcite, and monolithic aragonite. Both the cross-sectional nanoindentation measurements taken in this study, and previous reports on the bulk properties of the materials are shown[10-25]. Values from the literature are averages that represent each test type.

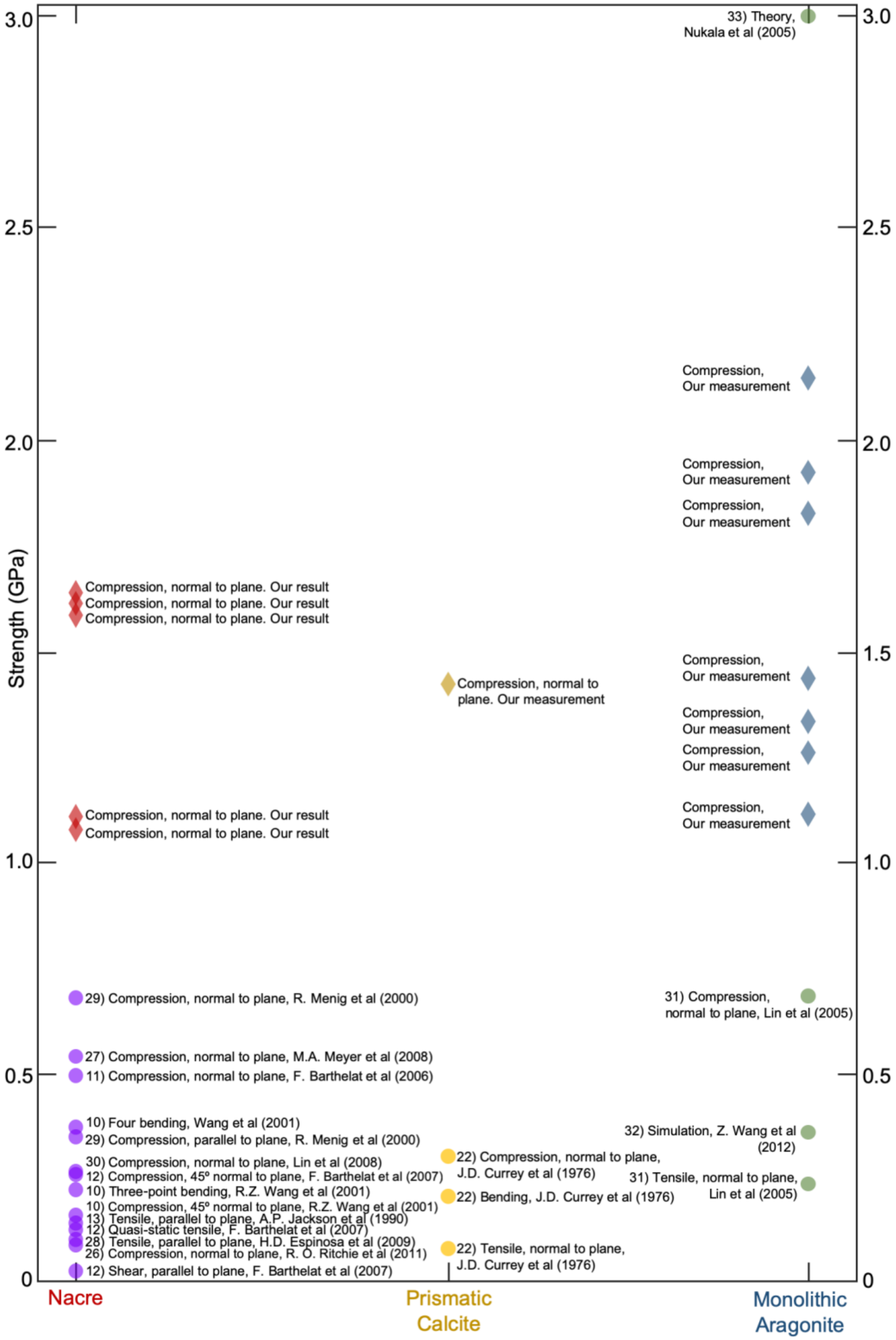


**Supplementary Figure 13.** Literature survey of strength of nacre, prismatic calcite, and monolithic aragonite. Both the cross-sectional nanoindentation measurements taken in this study, and previous reports on the bulk properties of the materials are shown[10-13,22,26-33]. Values from the literature are averages that represent each test type.

## References


1 Nudelman, F. Nacre biomineralisation: A review on the mechanisms of crystal nucleation. *Seminars in Cell & Developmental Biology* **46**, 2-10 (2015).

2 Checa, A. G., Cartwright, J. H. E. & Willinger, M.-G. Mineral bridges in nacre. *Journal of Structural Biology* **176**, 330-339 (2011).

3 Cartwright Julyan, H. E. & Checa Antonio, G. The dynamics of nacre self-assembly. *Journal of The Royal Society Interface* **4**, 491-504 (2007).

4 Suzuki, M. *et al.* An Acidic Matrix Protein, Pif, Is a Key Macromolecule for Nacre Formation. *Science* **325**, 1388-1390 (2009).

5 Shen, X., Belcher, A. M., Hansma, P. K., Stucky, G. D. & Morse, D. E. Molecular Cloning and Characterization of Lustrin A, a Matrix Protein from Shell and Pearl Nacre of Haliotis rufescens. *Journal of Biological Chemistry* **272**, 32472-32481 (1997).

6 Blank, S. *et al.* The nacre protein perlucin nucleates growth of calcium carbonate crystals. *Journal of Microscopy* **212**, 280-291 (2003).

7 Hovden, R. & Muller, D. A. Efficient elastic imaging of single atoms on ultrathin supports in a scanning transmission electron microscope. *Ultramicroscopy* **123**, 59-65 (2012).

8 Fischer, A. M. *et al.* Highly luminescent, high-indium-content InGaN film with uniform composition and full misfit-strain relaxation. *Applied Physics Letters* **103**, 131101 (2013).

9 MacDonald, G. J. F. Experimental determination of calcite-aragonite equilibrium relations at elevated temperatures and pressures. *American Mineralogist* **41**, 744-756 (1956).

10 Wang, R. Z., Suo, Z., Evans, A. G., Yao, N. & Aksay, I. A. Deformation mechanisms in nacre. *Journal of Materials Research* **16**, 2485-2493 (2001).

11 Barthelat, F., Li, C.-M., Comi, C. & Espinosa, H. D. Mechanical properties of nacre constituents and their impact on mechanical performance. *Journal of Materials Research* **21**, 1977-1986 (2006).

12 Barthelat, F., Tang, H., Zavattieri, P., Li, C. & Espinosa, H. On the mechanics of mother-of-pearl: A key feature in the material hierarchical structure. *Journal of the Mechanics and Physics of Solids* **55**, 306-337 (2007).

13 Jackson, A. P., Vincent, J. F. V. & Turner, R. M. Comparison of nacre with other ceramic composites. *Journal of Materials Science* **25**, 3173-3178 (1990).

14 Song, F., Soh, A. K. & Bai, Y. L. Structural and mechanical properties of the organic matrix layers of nacre. *Biomaterials* **24**, 3623-3631 (2003).

15 Okumura, K. & de Gennes, P. G. Why is nacre strong? Elastic theory and fracture mechanics for biocomposites with stratified structures. *The European Physical Journal E* **4**, 121-127 (2001).

16 Fitzer, S. C. *et al.* Ocean acidification alters the material properties of Mytilus edulis shells. *Journal of the Royal Society Interface* **12**, 20141227 (2015).

17 Li, X., Chang, W.-C., Chao, Y. J., Wang, R. & Chang, M. Nanoscale Structural and Mechanical Characterization of a Natural Nanocomposite Material: The Shell of Red Abalone. *Nano Letters* **4**, 613-617 (2004).

18 Stempflé, P., Pantalé, O., Rousseau, M., Lopez, E. & Bourrat, X. Mechanical properties of the elemental nanocomponents of nacre structure. *Materials Science and Engineering: C* **30**, 715-721 (2010).

19 Sun, J.-y. & Tong, J. Fracture toughness properties of three different biomaterials measured by nanoindentation. *Journal of Bionic Engineering* **4**, 11-17 (2007).

20 Kunitake, M. E., Mangano, L. M., Peloquin, J. M., Baker, S. P. & Estroff, L. A. Evaluation of strengthening mechanisms in calcite single crystals from mollusk shells. *Acta Biomaterialia* **9**, 5353-5359 (2013).
21 Merkel, C. *et al.* Mechanical properties of modern calcite- (Mergerlia truncata) and phosphate-shelled brachiopods (Discradisca stella and Lingula anatina) determined by nanoindentation. *Journal of Structural Biology* **168**, 396-408 (2009).
22 Currey, J. D. Mechanical properties of mother of pearl in tension. *Proc. R. Soc. Lond. B* **196**, 443-463 (1977).
23 Wegst, U. G. K. & Ashby, M. F. The mechanical efficiency of natural materials. *Philosophical Magazine* **84**, 2167-2186 (2004).
24 Liu, L.-g., Chen, C.-c., Lin, C.-C. & Yang, Y.-j. Elasticity of single-crystal aragonite by Brillouin spectroscopy. *Physics and Chemistry of Minerals* **32**, 97-102 (2005).
25 Kearney, C. *et al.* Nanoscale Anisotropic Plastic Deformation in Single Crystal Aragonite. *Physical Review Letters* **96**, 255505 (2006).
26 Ritchie, R. O. The conflicts between strength and toughness. *Nature Materials* **10**, 817-822 (2011).
27 Meyers, M. A., Chen, P.-Y., Lin, A. Y.-M. & Seki, Y. Biological materials: Structure and mechanical properties. *Progress in Materials Science* **53**, 1-206 (2008).
28 Espinosa, H. D., Rim, J. E., Barthelat, F. & Buehler, M. J. Merger of structure and material in nacre and bone – Perspectives on de novo biomimetic materials. *Progress in Materials Science* **54**, 1059-1100 (2009).
29 Menig, R., Meyers, M. H., Meyers, M. A. & Vecchio, K. S. Quasi-static and dynamic mechanical response of Haliotis rufescens (abalone) shells. *Acta Materialia* **48**, 2383-2398 (2000).
30 Lin, A. Y.-M. *Structural and functional biological materials : abalone nacre, sharp materials, and abalone foot adhesion*, UC San Diego, (2008).
31 Lin, A. & Meyers, M. A. Growth and structure in abalone shell. *Materials Science and Engineering: A* **390**, 27-41 (2005).
32 Wang, J., Cheng, Q. & Tang, Z. Layered nanocomposites inspired by the structure and mechanical properties of nacre. *Chemical Society Reviews* **41**, 1111-1129 (2012).
33 Nukala, P. K. V. V. & Simunovic, S. A continuous damage random thresholds model for simulating the fracture behavior of nacre. *Biomaterials* **26**, 6087-6098 (2005).
